# Impact of DNA integrity on the success rate of tissue‐based next‐generation sequencing: Lessons from nationwide cancer genome screening project SCRUM‐Japan GI‐SCREEN

**DOI:** 10.1111/pin.13029

**Published:** 2020-10-08

**Authors:** Takeshi Kuwata, Masashi Wakabayashi, Yutaka Hatanaka, Eiichi Morii, Yoshinao Oda, Kenichi Taguchi, Masayuki Noguchi, Yuichi Ishikawa, Takashi Nakajima, Shigeki Sekine, Shogo Nomura, Wataru Okamoto, Satoshi Fujii, Takayuki Yoshino, SCRUM‐Japan GI‐SCREEN Pathology Group

**Affiliations:** ^1^ Department of Genetic Medicine and Services National Cancer Center Hospital East Chiba Japan; ^2^ Department of Pathology and Clinical Laboratories National Cancer Center Hospital East Chiba Japan; ^3^ Clinical Research Support Office National Cancer Center Hospital East Chiba Japan; ^4^ Research Division of Genome Companion Diagnostics Hokkaido University Hospital Hokkaido Japan; ^5^ Department of Pathology Osaka University Graduate School of Medicine Osaka Japan; ^6^ Department of Anatomic Pathology, Pathological Sciences, Graduate School of Medical Sciences Kyushu University Fukuoka Japan; ^7^ Department of Pathology National Hospital Organization Kyushu Cancer Center Fukuoka Japan; ^8^ Department of Diagnostic Pathology, Faculty of Medicine University of Tsukuba Ibaraki Japan; ^9^ Department of Pathology, the Cancer Institute Japanese Foundation for Cancer Research Tokyo Japan; ^10^ Division of Diagnostic Pathology Shizuoka Cancer Center Hospital Shizuoka Japan; ^11^ Department of Pathology National Cancer Center Hospital Tokyo Japan; ^12^ Translational Research Support Section National Cancer Center Hospital East Chiba Japan; ^13^ Cancer Treatment Center Hiroshima University Hospital Hiroshima Japan; ^14^ Department of Molecular Pathology Yokohama City University Graduate School of Medicine Kanagawa Japan; ^15^ Department of Gastrointestinal Oncology National Cancer Center Hospital East Chiba Japan

**Keywords:** DNA integrity, formalin‐fixed paraffin‐embedded tumor tissue, next‐generation sequencing, quality control

## Abstract

In the nationwide cancer genome screening project SCRUM‐Japan GI‐SCREEN, 2590 archival formalin‐fixed paraffin‐embedded (FFPE) tumor tissues from 19 institutions were analyzed with two tissue‐based next‐generation sequencing (NGS) panels at the Clinical Laboratory Improvement Amendments (CLIA)‐certified College of American Pathologists (CAP)‐accredited central laboratory. The Oncomine Cancer Research Panel (OCP; 143 genes) succeeded in producing validated results for only 68.3% of the samples (%OCP‐success). CE‐IVD (25 genes) succeeded in 45.9% of the OCP‐failed samples, leading to an overall NGS success (%combined‐success) rate as high as 82.9%. Among 2573 samples, the DNA‐integrity (Δ*C*_t_)‐high (Δ*C*_t_ < 4.4, *n* = 1253) samples showed significantly higher %OCP‐ and %combined‐success rates (90.2% and 97.4%, respectively) than the DNA‐integrity‐intermediate (4.4 < Δ*C*_t_ < 6.3, *n* = 911; 68.9% and 88.7%) and DNA‐integrity‐low ones (Δ*C*_t_ > 6.3 or polymerase chain reaction‐failed, *n* = 409; 5.6% and 24.7%). Other factors associated with NGS success included the FFPE‐sample storage period (<4 years), the specimen type (surgical) and the primary tumor site (colorectal). Multivariable analysis revealed DNA integrity as the factor with the strongest independent association with NGS success, although it was suggested that other institution‐specific factors contribute to the discordance of inter‐institutional NGS success rates. Our results emphasize the importance of DNA quality in FFPE samples for NGS tests and the impact of DNA integrity on quality monitoring of pathology specimens for achieving successful NGS.

**Abbreviations**
CAP: College of American Pathologists
CLIA: Clinical Laboratory Improvement Amendments
CRC: colorectal cancer
*C*_t_: threshold cycle
EDC: electronic data capture
FFPE: formalin‐fixed paraffin‐embedded
GIST: gastrointestinal stromal tumor
H&E: hematoxylin and eosin
HCC: hepatocellular carcinoma
NBF: neutral buffered formalin
NET/NEC: neuroendocrine tumor/neuroendocrine carcinoma
NGS: next‐generation sequencing
OCP: Oncomine Cancer Research Panel
qPCR: quantitative polymerase chain reaction
QA: quality assurance
QC: quality control

## INTRODUCTION

In the era of precision oncology, the best‐matched therapy should be delivered to each cancer patient based on comprehensive cancer genome profiles obtained by next‐generation sequencing (NGS).^1^, ^2^, ^3^ Owing to recent technological improvements, archival formalin‐fixed paraffin‐embedded (FFPE) tumor tissues can be applied in routine pathological diagnosis using NGS tests.^4^ The potential clinical utility of evaluating comprehensive cancer genome profiles from FFPE tumor tissue samples has been suggested by several clinical trials.^5^, ^6^ To date, two NGS panels for advanced solid tumors as well as another small lung cancer panel have been approved in Japan as *in vitro* diagnostics. Because NGS is the most complex technology in molecular diagnostics, quality assurance (QA) and quality control (QC) must be applied throughout NGS procedures to ensure the accuracy of the test results.^7^, ^8^, ^9^ Importantly, the DNA quality of FFPE samples also needs to be assessed, as several modifications during the preparation of such samples potentially have adverse effects on the NGS analysis.^10^

In February 2015, we initiated the nationwide cancer genome screening project called SCRUM‐Japan GI‐SCREEN (known as MONSTAR‐SCREEN since April 2019).^11^, ^12^, ^13^ In this project, patients with advanced gastrointestinal cancer have been enrolled from 19 institutions across Japan, and their FFPE samples have been analyzed with two NGS panels at a single Clinical Laboratory Improvement Amendments (CLIA)‐certified College of American Pathologists (CAP)‐accredited central laboratory.

In this article, we present an investigation of the association between the DNA quality and the success rate of tissue‐based NGS analysis, using archival FFPE samples submitted from the institutions participating in SCRUM‐Japan GI‐SCREEN, to clarify an appropriate archival FFPE sample for NGS analyses for pathologists as well as treating physicians.

## MATERIALS AND METHODS

### Patient eligibility and FFPE sample preparation

The patients diagnosed with advanced gastrointestinal cancers and participating in SCRUM‐Japan GI‐SCREEN from February 2015 to April 2017 at 19 institutions (Table S1) were subjects for this study. The study protocol was approved by the institutional review board at each institution and written informed consent was obtained from each patient. Patients who withdrew their consent were excluded. This study was performed in accordance with the ethical standards laid down in the Declaration of Helsinki. The study was registered at UMIN Clinical Trials Registry (UMIN‐CTR; registration numbers UMIN000016343 and UMIN000016344). The clinicopathological data were collected through an electronic data capture (EDC) system. According to the definitions in the study protocol, neuroendocrine tumors/neuroendocrine carcinomas (NET/NEC), gastrointestinal stromal tumors (GIST), and appendix and anal canal cancers were classified as noncolorectal cancers (non‐CRC) even if they were of colorectal origin, while only colorectal adenocarcinoma was classified as CRC.

Basically, nine 7‐μm‐thick consecutive sections, as predetermined in the study protocol with the expectation of obtaining a sufficient amount of DNA/RNA for performing the Oncomine Cancer Research Panel (OCP)/CE‐IVD (20 ng each), even with small biopsy specimens, were prepared from single archival FFPE tumor tissue blocks for NGS analyses, along with an additional section for hematoxylin and eosin (H&E) staining. All of the pathology samples were examined and selected by local pathologists and prepared at the pathology laboratories of the participating institutions. The prepared sections were directly shipped from each institution to the CLIA‐certified CAP‐accredited central laboratory at ThermoFisher Scientific (West Sacramento, CA, USA) for DNA and RNA extraction, followed by NGS analyses.

### Tumor DNA/RNA extraction and DNA integrity determination

The presence of cancer lesions in the samples was confirmed by the central pathologists, working at the central laboratory, by examining the corresponding H&E‐stained sections at the central laboratory in the US. In cases in which there were any discordant pathological findings of the samples between the local pathologists of the participating institutions and the central pathologists of the central laboratory, the Japanese central pathologists (TK and SF) in the SCRUM‐Japan GI‐SCREEN examined the images as virtual slides and discussed them with the central pathologists in the US to make the final decision. Manual micro‐dissection was performed for tumor tissue enrichment as the minimum tumor content within the region of interest was at least 50%. Tumor DNA and RNA were extracted by using spin‐column methods using the RecoverAll Total Nucleic Acid Isolation Kit for FFPE (ThermoFisher Scientific, Waltham, MA, USA) and the columns in the PureLink RNA Micro Kit (ThermoFisher Scientific, Waltham) by eluting the DNA and RNA in 30 μL of elution buffer. DNA and RNA concentrations were measured using Qubit Assay Kit (ThermoFisher Scientific, Waltham). DNA integrity was determined by quantitative polymerase chain reaction (qPCR) using 3 ng of DNA with two sets of PCR primers for amplifying long (157 bp) and short (93 bp) target product sequences. The DNA integrity (Δ*C*_t_ value) was designated as follows:

$$\begin{array}{lll} \Delta Ct\; & = & \left[\text{threshold}\;\text{cycle}\;(Ct)\;\text{value}\;\text{for}\;\text{short}\;\text{PCR}\;\text{product}\right]\\ & & –\;[Ct\;\text{for}\;\text{long}\;\text{PCR}\;\text{product}]. \end{array}$$

If the *C*_t_ value for the short PCR product was over 34.5, Δ*C*_t_ would not be determined and a designation of ‘PCR‐failed’ was applied.

### Procedure of the NGS panel analysis and QC‐metrics

The OCP tests, which detect 143 gene alterations including single‐nucleotide variants, insertions and deletions, copy number variants and fusions, were performed using the extracted tumor DNA and RNA. The CE‐IVD tests, which detect 22 gene mutations and three translocations (ALK, RET and ROS1), were also performed together with the OCP either simultaneously or sequentially. Positive results regarding the detection of variants were confirmed in Integrative Genomic Viewer. After all analytical procedures had been completed, the obtained data were evaluated under the predetermined QC‐metrics, and reported as the validated results only if all the QC‐metrics were confirmed. When the procedures were designated as QC‐failure, the label ‘not analyzable’ was assigned. Information on the QC‐metrics established by ThermoFisher Scientific, including RUN QC and sample QC, is provided in Table S2. There are equivalent levels of stringency in the QC‐metrics in the OCP and CE‐IVD panels. If both OCP and CE‐IVD panels produced the validated results, the OCP results were selected as the final ones. The proportion of the samples from which the OCP panel analyses met the predesigned QC‐metrics and produced the validated results was defined as the %OCP‐success rate. The proportion of the samples in either OCP or CE‐IVD panel analyses that produced validated results was defined as the %combined‐success rate. In this study, we investigated the QC‐metrics for only DNA but not RNA analyses.

### Initial pilot phase and subsequent expansion phase

Initial pilot phase assessment was conducted to confirm the clinical performance of the OCP and to sequentially perform CE‐IVD tests for the samples for which OCP failed at three institutions for CRC within 3 months after the enrollment of the first patient. Clinical performance in both panel tests and Δ*C*_t_ values in each sample were reviewed, to develop the sample submission algorithm in the subsequent expansion phase for all advanced gastrointestinal cancers in all participating institutions.

### Statistical analysis

Clopper and Pearson's method was used for calculating 95% confidence intervals (CI) of %OCP‐success and %combined‐success rates, and Pearson's *χ* ^2^ test was used for their comparisons. Univariable and multivariable logistic regression models were used to assess the association between the NGS success rates (%OCP‐success and %combined‐success rates) and the following factors: DNA integrity (>6.3 or qPCR‐failed/>4.4, <6.3/<4.4), submitted institution (ID 02‐19/ID 01), sex (female/male), age (>50 years/<50 years), specimen type (surgical/biopsy), site of specimen (metastatic/primary), histology (nonadenocarcinoma/adenocarcinoma), primary tumor site (noncolorectal/colorectal), FFPE‐sample storage period (>4 years/<4 years), previous chemotherapy (received/not received) and previous radiotherapy (received/not received). The *P*‐values were reported as two‐sided, and *P* < 0.05 was considered to be statistically significant. All statistical analyses were performed using SAS 9.4 software (SAS Institute, Cary, NC, USA).

## RESULTS

### Initial pilot phase results for developing the sample submission algorithm

**Figure 1:**
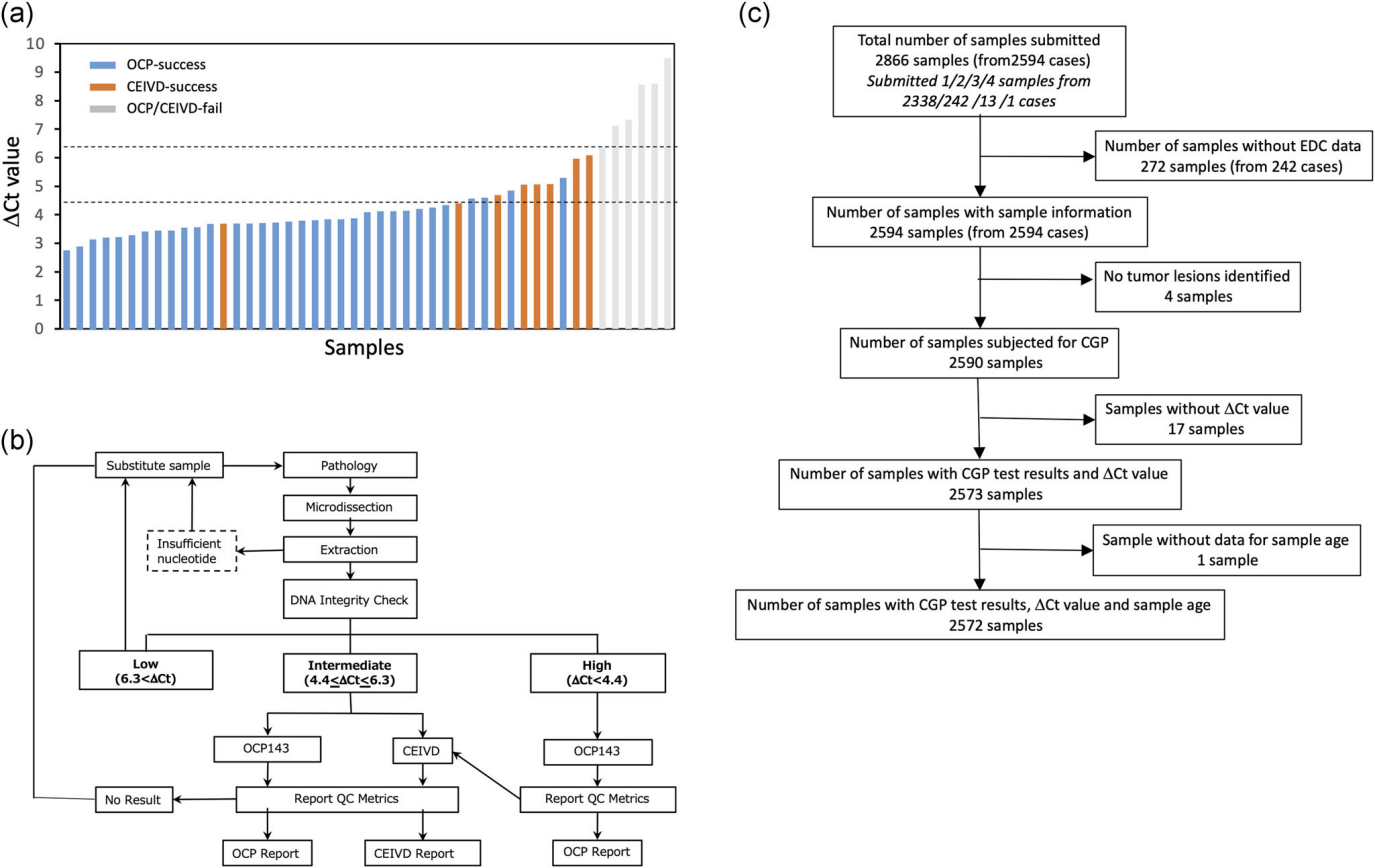
(**a**) Distribution of DNA integrity in initial 47 samples with Δ*C*_t_ (threshold cycle) values and their results with Oncomine Cancer Research Panel (OCP) and CI‐IVD analyses. Blue, OCP‐success; Orange, CE‐IVD‐success; Gray, both OCP and CE‐IVD failed. (**b**) Algorithm for sample submission and NGS panel selection used in SCRUM‐Japan GI‐SCREEN. The DNA extracted from FFPE tissue samples was examined for DNA integrity (Δ*C*_t_). If the DNA integrity was Δ*C*_t_ < 4.4 (high), the DNA would be applied for OCP analysis. If the DNA integrity was 4.4 < Δ*C*_t_ < 6.3 (intermediate), the DNA was applied for both OCP and CE‐IVD simultaneously. If the DNA integrity was Δ*C*_t_ > 6.3 (low), the samples would either be proceed or be replaced by other samples, at the investigator's discretion. Additionally, if the initial analysis failed, sample re‐submission would be allowed until the final NGS results were obtained using either OCP or CE‐IVD panel. (**c**) Consort diagram of samples and patients analyzed in this study.

In the initial pilot phase assessment, the results of 66 FFPE CRC tissue samples were reviewed. The OCP tests were successful in producing the validated results from only 49 samples (%OCP‐success rate, 74.2%); for 17 samples (25.8%), validated results could not be obtained due to QC failure. Re‐analyses of these 17 samples with CE‐IVD could produce validated results for eight samples. As such, by employing OCP and CE‐IVD in combination, the %combined‐success rate reached 86.4% (57/66). Among the 66 samples, the DNA integrity was measured in 47 samples. For almost all of the samples with a Δ*C*_t_ value under 4.4 (Δ*C*_t_ < 4.4), the validated results could be successfully produced with OCP (%OCP‐success 94.0%, 29/30), while OCP succeeded for only 23.5% (4/17) of the samples with Δ*C*_t_ > 4.4 (Fig. 1a). CE‐IVD could produce the validated results with all samples with Δ*C*_t_ < 6.3 (8/8), but for none of them with Δ*C*_t_ > 6.3 (0/6). Considering these results, we categorized the quality of FFPE samples based on the DNA integrity: high (Δ*C*_t_ < 4.4), intermediate (4.4 < Δ*C*_t_ < 6.3) or low (Δ*C*_t_ > 6.3 or PCR‐failed). On the basis of the DNA integrity level, a sample submission algorithm was developed (Fig. 1b).

### Clinicopathological characteristics and NGS success rates of the overall samples

From February 2015 to April 2017, a total of 2594 patients, including the patients who participated in the initial pilot phase, were enrolled. Among them, more than two FFPE samples were submitted from 242 patients and a total of 2866 samples were examined (Fig. 1c). The clinicopathological characteristics of the 2594 patients and their samples as assigned by the NGS results are listed in Table 1. Overall, 1001 of the patients had CRC and 1593 had another condition. Approximately half of the samples were surgical specimens (54.6%) and 85.2% of the samples were obtained from primary sites. In terms of the histological type, the majority of cases were adenocarcinoma (85.1%). More than 90% of samples had been prepared within 4 years (92.2%). Four samples with no tumor lesion were excluded from further analyses, thus data on the NGS analyses were available for the remaining 2590 samples. Overall, the %OCP‐success rate was 68.3% (1769/2590). CE‐IVD succeeded in producing validated results from 45.9% (377/821) of the Samples for which OCP failed, leading to a %combined‐success rate as high as 82.9% (2146/2590).

**Table 1:** Clinicopathological characteristics

	Number (%, total 2594 cases)
Sex (male/female)	1682/912 (64.8/35.2)
Age (≤50 years/>50 years)	325/2269 (12.5/87.5)
Primary tumor site	
Colorectal	1001 (38.6)
Noncolorectal	1593 (61.4)
Stomach	743 (28.6)
Esophagus	232 (8.9)
Pancreas	247 (9.5)
Biliary tract	163 (6.3)
Liver (HCC)	49 (1.9)
Others	71 (2.7)
* * GIST	55 (2.1)
NET/NEC	33 (1.3)
Specimen type (surgery/biopsy)	1420/1174 (54.6/45.4)
Site of obtained specimen (primary/metastatic)	2209/385 (85.2/14.8)
Histology	
Adenocarcinoma	2207 (85.1)
Nonadenocarcinoma	387 (14.9)
Squamous cell carcinoma	224 (8.6)
Gastrointestinal stromal tumor	55 (2.1)
Hepatocellular carcinoma	49 (1.9)
NET/NEC	41 (1.6)
Adenosquamous cell carcinoma	5 (0.2)
Carcinoma, NOS/NA	13 (0.5)
Differentiation grade	
Well/moderately differentiated	1658 (63.9)
Poorly differentiated	579 (22.3)
Mucinous	52 (2.0)
NEC/NET	41 (1.6)
Not available	262 (10.1)
FFPE‐sample storage period (<4 years, ≥4 years)	2390/203 (92.2/7.8)†
Previous chemotherapy (not received/received)	2042/552 (78.7/21.3)
Previous radiotherapy (not received/received)	2525/69 (97.3/2.7)

^†^ One patient for whom the information was missing was excluded.

FFPE, formalin‐fixed paraffin‐embedded; GIST, gastrointestinal stromal tumor; HCC, hepatocellular carcinoma; NET/NEC, neuroendocrine tumor/neuroendocrine carcinoma.

In the following investigations, excluding four samples with no tumor lesion and 17 samples with no Δ*C*_t_ value, we focused on the remaining 2573 FFPE samples (Fig. 1c).

### Impact of DNA integrity on the NGS‐panel success rate

First, we evaluated the associations of DNA integrity with %OCP‐ and %combined‐success rates with the overall 2573 samples. The %OCP‐ and %combined‐success rates in DNA‐integrity‐high samples were 90.2% (95% CI 88.4–91.8%) and 97.4% (95% CI 96.4–98.2%), respectively (Table 2). In contrast, these rates were 5.6% (95% CI 3.6–8.3%) and 24.7% (95% CI 20.6–29.2%), respectively, in DNA‐integrity‐low samples, while the values for DNA‐integrity‐intermediate samples were between those of the above samples (68.9% (95% CI 62.7–68.9%) and 88.7% (95% CI 86.5–90.7%)).

**Table 2:** The %OCP‐ and %combined‐success rates by DNA integrity level

DNA integrity (Δ*C*_t_)	*n*	%OCP‐success		%Combined‐success	
		*n*	% (95% CI)	*n*	% (95% CI)
High (<4.4)	1253	1130	90.2 (88.4–91.8)	1221	97.4 (96.4–98.2)
Intermediate (≥4.4, ≤6.3)	911	600	68.9 (62.7–68.9)	808	88.7 (86.5–90.7)
Low (>6.3 or qPCR‐failed)	409	23	5.6 (3.6–8.3)	101	24.7 (20.6–29.2)

CI, confidence interval; *C*_t_, threshold cycle; OCP, Oncomine Cancer Research Panel; qPCR, quantitative polymerase chain reaction.

Using the univariable logistic regression model, DNA integrity was significantly related to the %OCP‐success rate (Table 3); the odds ratio (OR) of DNA‐integrity‐intermediate to DNA‐integrity‐high was 0.210 (95% CI 0.167–0.265, *P* < 0.0001) and that of DNA‐integrity‐low to DNA‐integrity‐high was 0.006 (95% CI 0.004–0.010, *P* < 0.0001). Furthermore, multivariable analyses adjusted by putative clinicopathological factors showed a significant association between DNA integrity and %OCP‐success rate: OR of DNA‐integrity‐intermediate to DNA‐integrity‐high was 0.215 (95% CI 0.169–0.272, *P* < 0.0001) and that of DNA‐integrity‐low to DNA‐integrity‐high was 0.008 (95% CI 0.005–0.012) (Table 3). Additionally, DNA integrity was also significantly associated with %combined‐success rate in univariable and multivariable analyses (Table S3). Thus, we concluded that DNA integrity is an indicator of the quality of FFPE samples for predicting the success of NGS.

**Table 3:** Clinicopathological factors associated with %OCP‐success rates

	Univariable (*n* = 2573)		Multivariable (*n* = 2572)	
	Odds ratio (95% CI)	*P*‐value	Odds ratio (95% CI)	*P*‐value
DNA integrity (intermediate/high)	**0.210 (0.167–0.265)**	**<0.0001**	**0.215 (0.169–0.272)**	**<0.0001**
DNA integrity (low/high)	**0.006 (0.004–0.010)**	**<0.0001**	**0.008 (0.005–0.012)**	**<0.0001**
Sex (female/male)	**1.198 (1.005–1.427)**	**0.0441**	**1.048 (0.831–1.322)**	**0.6933**
Age (>50 years/≤50 years)	0.906 (0.703–1.168)	0.4484	1.135 (0.820–1.572)	0.4448
Specimen type (surgical/biopsy)	**1.542 (1.305–1.821)**	**<0.0001**	**1.532 (1.205–1.946)**	**0.0005**
Site of obtained specimen (metastatic/primary)	1.054 (0.834–1.334)	0.6584	1.037 (0.749–1.435)	0.8288
Histology (nonadenocarcinoma/adenocarcinoma)	**0.663 (0.530–0.829)**	**0.0003**	0.837 (0.610–1.150)	0.2723
Primary tumor site (noncolorectal/colorectal)	**0.580 (0.486–0.692)**	**<0.0001**	**0.739 (0.574–0.951)**	**0.0188**
FFPE‐sample storage period (≥4 years/<4 years)†	**0.215 (0.158–0.290)**	**<0.0001**	**0.432 (0.291–0.642)**	**<0.0001**
Previous chemotherapy (received/not received)	**1.252 (1.017–1.541)**	**0.0338**	1.062 (0.794–1.421)	0.6837
Previous radiotherapy (received/not received)	0.933 (0.562–1.550)	0.789	0.827 (0.418–1.634)	0.5842

*Note*: Bold emphasis are used when *P* < 0.05.

^†^ One case without records was excluded for the univariable analysis.

CI, confidence interval; FFPE, formalin‐fixed paraffin‐embedded; OCP, Oncomine Cancer Research Panel.

### Clinicopathological factors associated with NGS‐panel success

In addition to DNA integrity, as shown in Table 3, univariable analysis revealed the following six clinicopathological factors as being significantly favorable for %OCP‐success: sex (female), specimen type (surgical specimen), histology (adenocarcinoma), primary tumor site (colorectal), FFPE‐sample storage period (<4 years), and previous chemotherapy (received). These same six factors were also associated with %combined‐success rate (Table S3). According to multivariable analysis, including all of the covariates used in the univariable analysis, the FFPE‐sample storage period (<4 years) along with the specimen type (surgical specimen) and primary tumor site (CRC) remained as independent factors significantly associated with %OCP‐success rate as well as %combined‐success rate, in addition to DNA integrity (Table 3 and Table S3).

**Figure 2:**
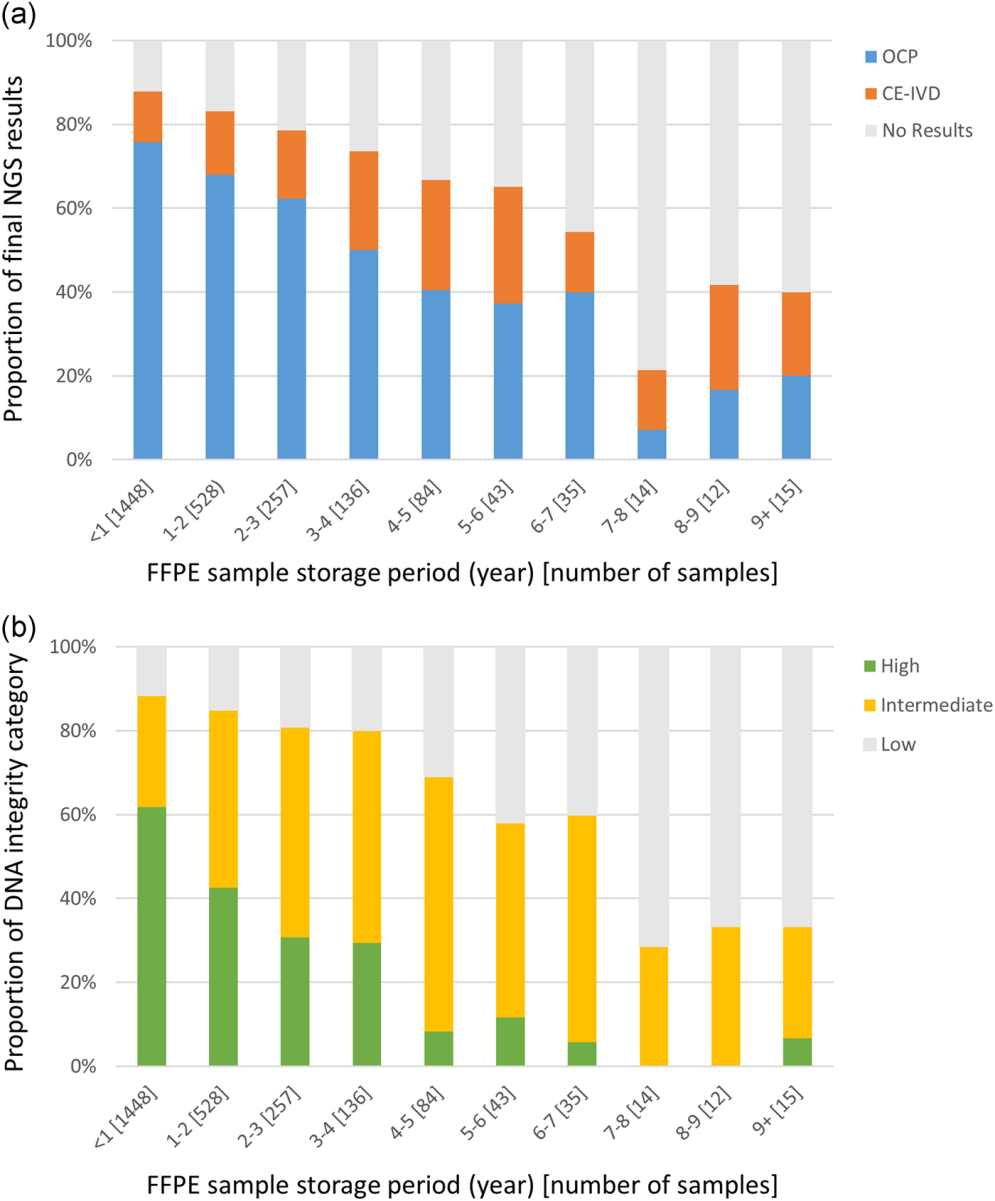
Influence of FFPE‐sample storage period on Oncomine Cancer Research Panel (OCP) and CE‐IVD panel results (**a**) and DNA integrity (**b**). The proportion of Δthreshold cycle (Δ*C*_t_) < 4.4 and the OCP‐success rate decreased in accordance with the formalin‐fixed paraffin‐embedded (FFPE)‐sample storage period.

Fig. 2(a) highlights the influence of the FFPE‐sample storage period on the NGS success rates. The %OCP‐success rate continuously decreased in accordance with the FFPE‐sample storage period and declined to 50% in 4 years. Simultaneously, the FFPE‐sample storage period influenced the DNA integrity (Fig. 2b), suggesting that the DNA in stored FFPE tissue samples continuously deteriorated. The quality of DNA in FFPE samples, represented as the DNA‐integrity‐high proportion, decreased more steeply than the %OCP‐success rate and dropped to less than 50% in 2 years.

**Figure 3:**
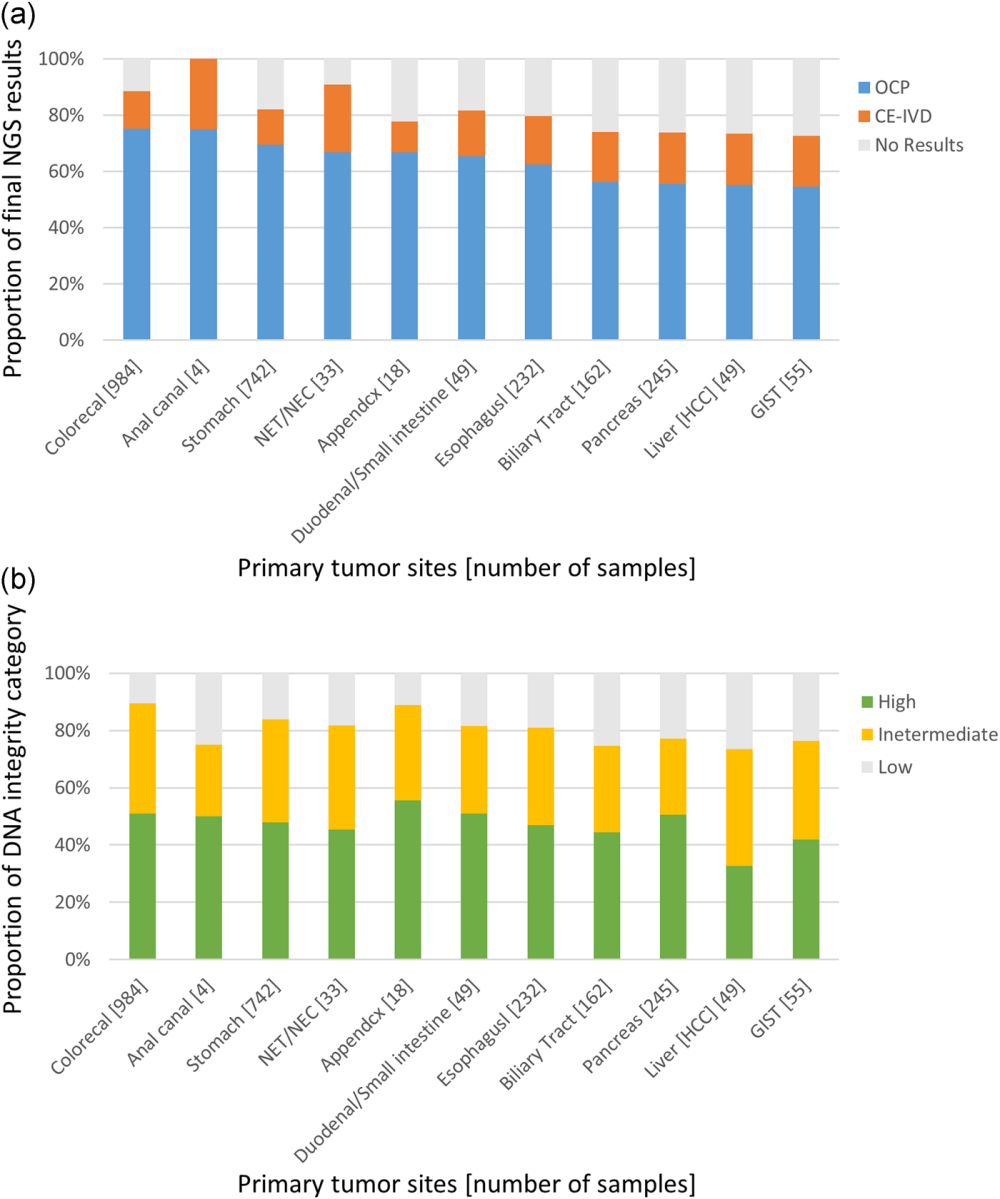
Influence of primary tumor site on Oncomine Cancer Research Panel (OCP) and CE‐IVD panel results (**a**) and DNA integrity (**b**). Basically, tumors originating from the alimentary tract showed a better success rate for OCP and a higher frequency of high DNA integrity (Δthreshold cycle (Δ*C*_t_) < 4.4).

The CRC patients showed a significantly higher %OCP‐success rate than patients with other conditions (75.2% vs. 63.8%). As shown in Fig. 3(a), those with hepatocellular carcinoma (HCC) and GIST showed the worst %OCP‐success rates (55.1% and 54.5%, respectively), consisting of the smallest DNA‐integrity‐high proportions (32.7% and 41.8%, respectively; Fig. 3b) among the subtypes.

Surgical specimens showed significantly higher %OCP‐success rates than biopsy specimens (63.0% and 72.4%, respectively). Concomitantly, the proportion of DNA‐integrity‐high/intermediate samples was higher in surgical than in biopsy specimens (87.7% and 79.8%, respectively; Fig. S1).

### Inter‐institutional discordance of NGS‐success rates

**Figure 4:**
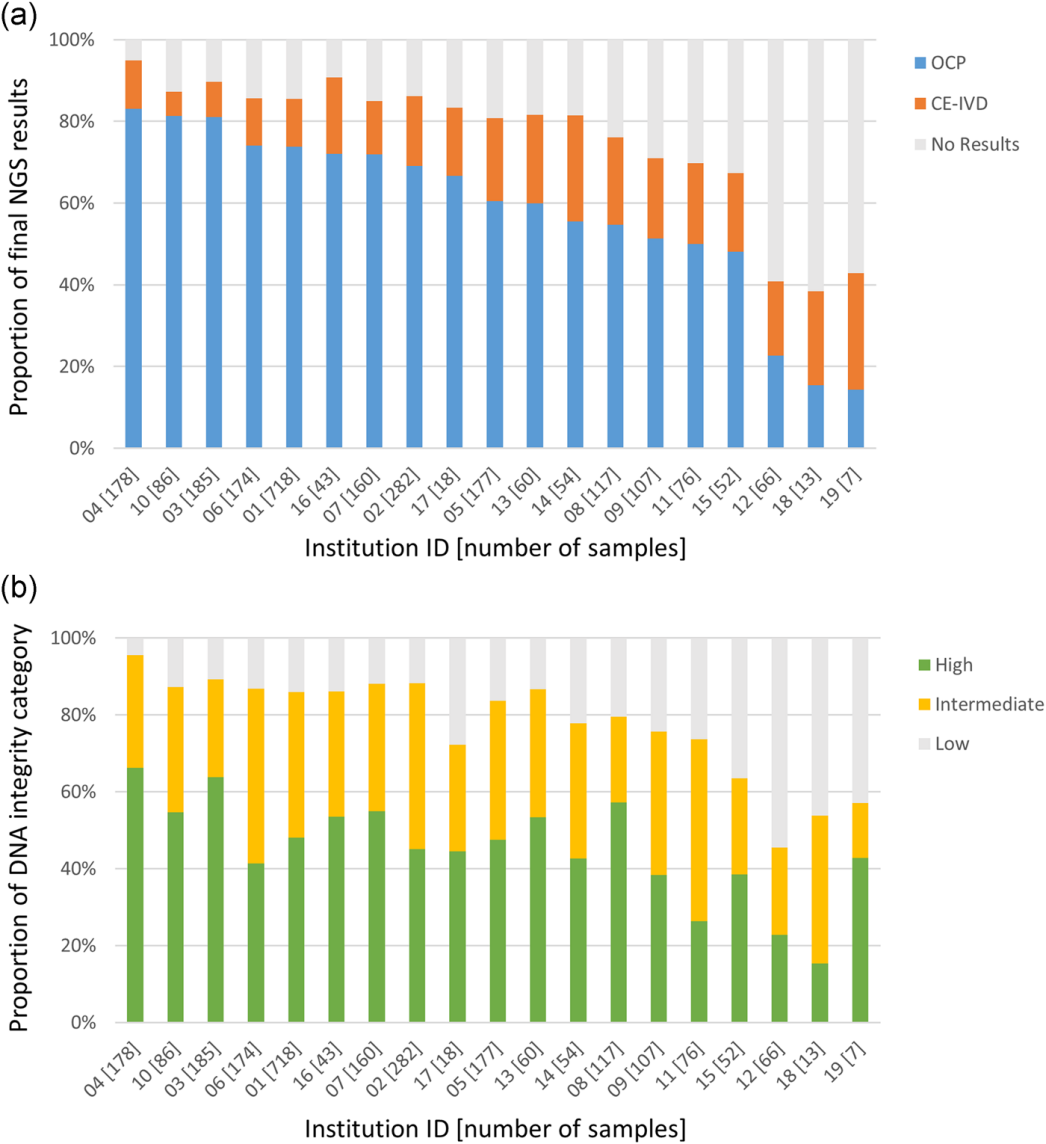
Proportions of Oncomine Cancer Research Panel (OCP)‐ and CE‐IVD‐success (**a**) and DNA integrity (**b**) among the institutions participating in SCRUM‐Japan GI‐SCREEN. Please note that the institutional IDs listed in this figure are not concordant with the order listed in Table S1.

Fig. 4(a) shows the proportions of samples for which OCP and CE‐IVD were successful in the 19 institutions. There were striking discordances in %OCP‐success (14.3–83.1%) and %combined‐success rates (38.5–94.9%) among the participating institutions. These discordances were statistically significant for both %OCP‐ and %combined‐success (Tables S4 and S5). The proportions of DNA‐integrity‐high (15.4–66.3%) and DNA‐integrity‐high/intermediate samples (45.5–95.5%) were also inconsistent among the institutions (Fig. 4b). To investigate the factors underlying the inter‐institutional discordance in NGS success rates, multivariable analyses were performed including the factor of the institution at which the sample was submitted as an additional putative covariate. The DNA integrity (for both %OCP‐ and %combined‐success rates) and FFPE‐sample storage period (for %OCP‐success rates) remained as independent factors strongly associated with %OCP‐ and %combined‐success rates (*P* < 0.0001, Tables S6 and S7). The factor of the institution at which the sample was submitted was also considered as an independent factor associated with %OCP‐ and %combined‐success rates in the analyses, suggesting that there may be additional institution‐specific factors contributing to the inter‐institutional discordance of NGS success rates.

## DISCUSSION

Next‐generation sequencing is the most reliable approach for revealing comprehensive genomic alterations in the era of precision oncology.^1^, ^3^, ^4^, ^14^ Recently developed technologies, especially NGS panels with selected cancer‐related genes, can examine a small amount of DNA/RNA extracted from biopsy samples.^2^, ^6^, ^14^, ^15^ However, it is extremely important to apply the QA/QC process throughout NGS procedures to obtain reliable results in a clinical setting.^7^, ^8^, ^9^, ^16^ In this study, all NGS analyses were performed in a single CLIA‐certified CAP‐accredited central laboratory and the obtained data were verified by the predetermined QC‐metrics. Although there is equivalent stringency in QC‐metrics between the two NGS panels, the smaller panel, CE‐IVD, allowed a larger read share for variant detection and showed a higher success rate than OCP.

During FFPE sample preparation, formalin‐induced DNA modifications in particular influence the efficacy of PCR and interfere with the library construction in NGS procedures. In contrast to commonly used DNA quantification methods, qPCR methods have an advantage of evaluating concomitant modifications and/or contamination interfering with PCR reactions.^17^, ^18^, ^19^ In this study, as real‐world data with 2573 archival FFPE samples from 19 institutions, we show that DNA integrity is significantly associated with the NGS success rate. Samples with poor DNA integrity also cause false‐positive mutation calls.^20^ Therefore, DNA integrity could be an excellent indicator for monitoring the quality of DNA in FFPE samples in the era of precision medicine.

Previously, Guyard *et al*. reported that DNA degradation in FFPE samples became evident after 4–6 years of storage.^21^ In this study, we observed deterioration of the OCP success rate in an even shorter storage period: the %OCP‐success rate and DNA‐integrity‐high proportion declined to 50% in 4 and 2 years of storage, respectively. Therefore, it would be advisable to select more recent FFPE samples for NGS analyses, especially for larger NGS panels.

In addition to the DNA integrity, the FFPE‐sample storage period, and other factors identified in this study, multivariable analysis including the factor of the institution at which the sample was submitted as a putative covariate indicated that there may be additional institution‐specific factors not identified in this study but contributing to the inter‐institutional discordance of NGS success rates. One possibility is the variation of formalin fixation conditions among the institutions. For immunohistochemistry, several factors including the formulation of fixatives and the duration of fixation influence the obtained results and are defined in the several guidelines.^22^, ^23^, ^24^ Unfortunately, information on the fixation conditions corresponding to each FFPE sample was not collected in SCRUM‐Japan GI‐SCREEN. However, it should be noted that, as of February 2016, such information was available for 16 institutions and variable formulations of formalin fixatives were used; only 75.0% (12/16) and 56.3% (9/16) of the institutions used 10% neutral‐buffered formalin (NBF) for biopsy and surgical specimens, respectively. Strikingly, for surgical specimens, all except one of the five institutions with the highest %NGS success rates used 10% NBF, while none (0%) of the five institutions with the lowest %NGS success rates did. Similarly, all of the five institutions with the highest %NGS success rates used 10% NBF for biopsy samples, but only two out of five institutions (40%) with the lowest %NGS success rates did. We also speculate that there may be other variables contributing to the inter‐institutional variation, such as warm/cold ischemic time and the proportion of samples not prepared in the participating themselves institutions but collected from the referral hospitals.

This study had limitations. First, as already explained, the influence of fixation conditions was not assessed in this study. Second, both OCP and CE‐IVD are amplicon‐sequencing‐based NGS panels. There may be other factors influencing the success of NGS on different platforms, such as hybridization‐capture‐based NGS panels. Third, only the results of DNA‐based analyses were evaluated in this study. The conditions may differ for RNA‐based NGS panels.^25^, ^26^

In conclusion, we identified DNA integrity as an excellent indicator for qualifying FFPE tissue samples considered for NGS analyses. Other results, especially the influence of FFPE‐sample storage period on the rate of NGS success, help pathologists as well as clinicians to practice precision oncology.

## DISCLOSURE STATEMENT

TK received research funds from Daiichi Sankyo Co. Ltd and Ono Pharm Co. Ltd. YH received lecture fees from AstraZeneca and Novartis, and research funds from ThermoFisher Scientific. TY received lecture fees from Bayer Yakuhin Ltd, Chugai Paharm Co. Ltd, Eli Lilly Japan K.K., Taiho Pharm. Co. Ltd and Merck Biopharma Co. Ltd and research funds from Amgen K.K., Daiichi Sankyo Co. Lt., MSD K.K., Ono Pharm. Co. Ltd, Parexel Int'l Inc. and Taiho Pharm. Co. Ltd.

## AUTHOR CONTRIBUTIONS

TK and TY conceived and designed the study. TK, YH, EM, YO, KT, MN, TN, SS and SF acquired and interpreted the pathological data. MN and MW examined and performed statistical analysis. TK and TY edited and reviewed the manuscript. All authors gave full approval for publication. TK takes full responsibility for the work.

## Supporting information

Additional Supporting Information may be found in the online version of this article at the publisher's website.

Supporting information.
