# Supplementary material for: Impact of DNA integrity on the success rate of tissue‐based next‐generation sequencing: Lessons from nationwide cancer genome screening project SCRUM‐Japan GI‐SCREEN
Source: Pathol Int. 2020 Oct 8;70(12):932–42. doi: 10.1111/pin.13029 (PMC7820973; doi:10.1111/pin.13029)

**Table S1  List of the 19 institutions participating in SCRUM-Japan GI-SCREEN during the study period**

Aichi Cancer Center Hospital
Cancer Institute Hospital of JFCR
Chiba Cancer Center
Hokkaido University Hospital
Kanagawa Cancer Center
Kanazawa University Hospital
Kansai Rosai Hospital
Keio University Hospital
Kindai University Hospital
Kyorin University Hospital
Kyushu Cancer Center
Kyushu University Hospital
National Cancer Center Hospital
National Cancer Center Hospital East
Osaka University Hospital
Saitama Cancer Center, Chiba Cancer Center
Shikoku Cancer Center
St. Marianna University School of Medicine Hospital
Tsukuba University Hospital

**Table S2 QC-metrics established in the central laboratory for OCP/CE-IVD tests**

| Metric | Criteria |
|---|---|
| **Run QC** | |
| DNA NTC | Read length histogram evaluated to ensure no contamination in the run and IR review of variant level data |
| RNA NTC | Read length histogram evaluated to ensure no contamination in the run and IR review of variant level data |
| Mappable Fusion Reads | >20,000 |
| **Sample QC - DNA Only** | |
| Uniformity | 85% |
| Mean Depth | >1000 |
| **DNA Control** | |
| BRAF p.Val600Glu | Present in IR and >5% AF |
| EGFR p.Glu746_Ala750del | Present in IR and >5% AF |
| KRAS p.Gly12Phe | Present in IR and >5% AF |
| **RNA Control** | |
| EML4/ALK | Present in IR |

**Table S3 Clinicopathological factors associated with %combined-success rates**

| | Univariable (n=2573) | | Multivariable (n=2572) | |
|---|---|---|---|---|
| | Odds (95% CI) | P-value | Odds (95% CI) | P-value |
| DNA integrity (intermediate/high) | **0.206 (0.137–0.309)** | **<0.0001** | **0.211 (0.140–0.319)** | **<0.0001** |
| DNA integrity (low/high) | **0.009 (0.006–0.013)** | **<0.0001** | **0.010 (0.007–0.015)** | **<0.0001** |
| Sex (female/male) | **1.246 (1.000–1.552)** | **0.0495** | 1.164 (0.858–1.578) | 0.3301 |
| Age (>50 years/≤50 years) | 0.961 (0.703–1.313) | 0.8014 | 1.397 (0.914–2.136) | 0.1220 |
| Specimen type (surgical/biopsy) | **1.667 (1.356–2.049)** | **<0.0001** | **1.386 (1,015–1.894)** | **0.0402** |
| Site of obtained specimen (metastatic/primary) | 0.954 (0.717–1.268) | 0.7433 | 0.850 (0.562–1.288) | 0.4439 |
| Histology (non-adenocarcinoma/adenocarcinoma) | **0.709 (0.543–0.925)** | **0.0114** | 1.076 (0.724–1.600) | 0.7171 |
| Primary tumor site (non-colorectal/colorectal) | **0.495 (0.393–0.623)** | **<0.0001** | **0.615 (0.441–0.858)** | **0.0042** |
| FFPE-sample storage period (≥4 years/<4 years)† | **0.241 (0.178–0.325)** | **<0.0001** | **0.510 (0.329–0.790)** | **0.0025** |
| Previous chemotherapy (received/not received) | **1.439 (1.099–1.884)** | **0.0082** | 1.244 (0.836–1.851) | 0.2822 |
| Previous radiotherapy (received/not received) | 1.099 (0.572–2.112) | 0.7762 | 0.924 (0.368–2.322) | 0.8665 |

†One case with no records was excluded from univariable analysis.

**Table S4 %OCP-success rates in the participating institutions**

| Institution ID | n | %OCP-success (95% CI) | Univariable (n=2573) | | Multivariable (n=2572) | |
|---|---|---|---|---|---|---|
| | | | Odds (95% CI) | P-value | Odds (95% CI) | P-value |
| 01 | 718 | 73.8% (70.4–77.0%) | ref. | (<0.0001) | ref. | (<0.0001) |
| 02 | 282 | 69.2% (63.4–74.5%) | 0.795 (0.587–1.076) | 0.1374 | **0.543 (0.366–0.805)** | **0.0024** |
| 03 | 185 | 81.1% (74.7–86.5%) | **1.520 (1.015–2.277)** | **0.0420** | 1.156 (0.664–2.014) | 0.6080 |
| 04 | 178 | 83.2% (76.8–88.3%) | **1.750 (1.143–2.680)** | **0.0101** | 0.740 (0.436–1.254) | 0.2628 |
| 05 | 177 | 60.5% (52.8–67.7%) | **0.542 (0.384–0.765)** | **0.0005** | **0.351 (0.225–0.547)** | **<0.0001** |
| 06 | 174 | 74.1% (67.0–80.5%) | 1.017 (0.697–1.484) | 0.9309 | 0.883 (0.538–1.448) | 0.6211 |
| 07 | 160 | 71.9% (64.2–78.7%) | 0.906 (0.618–1.329) | 0.6151 | **0.562 (0.340–0.930)** | **0.0249** |
| 08 | 117 | 54.7% (45.2–63.9%) | **0.428 (0.287–0.639)** | **<0.0001** | **0.208 (0.122–0.357)** | **<0.0001** |
| 09 | 107 | 51.4% (41.5–61.2%) | **0.375 (0.248–0.568)** | **<0.0001** | **0.284 (0.164–0.492)** | **<0.0001** |
| 10 | 86 | 81.4% (71.6–89.0%) | 1.552 (0.879–2.739) | 0.1294 | 1.454 (0.671–3.151) | 0.3429 |
| 11 | 76 | 50.0% (38.3–61.7%) | **0.355(0.220–0.573)** | **<0.0001** | **0.430 (0.233–0.792)** | **0.0068** |
| 12 | 66 | 22.7% (13.3–34.7%) | **0.104 (0.057–0.190)** | **<0.0001** | **0.116 (0.053–0.258)** | **<0.0001** |
| 13 | 60 | 60.0% (46.5–72.4%) | **0.532 (0.309–0.915)** | **0.0227** | **0.228 (0.115–0.452)** | **<0.0001** |
| 14 | 54 | 55.6% (41.4–69.1%) | **0.443 (0.253–0.778)** | **0.0046** | **0.237 (0.111–0.502)** | **0.0002** |
| 15 | 52 | 48.1% (34.0–62.4%) | **0.328 (0.186–0.580)** | **0.0001** | **0.367 (0.159–0.845)** | **0.0184** |
| 16 | 43 | 72.1% (56.3–84.7%) | 0.916 (0.461–1.821) | 0.8031 | 0.599 (0.244–1.472) | 0.2641 |
| 17 | 18 | 66.7% (41.0–86.7%) | 0.709 (0.263–1.917) | 0.4985 | 1.178 (0.238–5.830) | 0.8413 |
| 18 | 13 | 15.4% (1.9–45.5%) | **0.064 (0.014–0.294)** | **0.0004** | **0.058 (0.009–0.349)** | **0.0019** |
| 19 | 7 | 14.3% (0.4–57.9%) | **0.059 (0.007–0.494)** | **0.0090** | **0.016 (0.001–0.175)** | **0.0007** |

**Table S5 %Combined-success rates in the participating institutions**

| Institution ID | n | %OCP-success (95% CI) | Univariable (n=2573) | | Multivariable (n=2572) | |
|---|---|---|---|---|---|---|
| | | | Odds (95% CI) | P-value | Odds (95% CI) | P-value |
| 01 | 718 | 85.5% (82.7–88.0%) | ref | (<0.0001) | ref | (0.0002) |
| 02 | 282 | 86.2% (81.6–90.0%) | 1.055 (0.710–1.570) | 0.7901 | 0.866 (0.512–1.465) | 0.5909 |
| 03 | 185 | 89.7% (84.4–93.7%) | 1.480 (0.881–2.485) | 0.1382 | 1.220 (0.610–2.442) | 0.5739 |
| 04 | 178 | 94.9% (90.6–97.7%) | **3.181 (1.576–6.417)** | **0.0012** | 1.593 (0.675–3.759) | 0.2879 |
| 05 | 177 | 80.8% (74.2–86.3%) | 0.712 (0.464–1.093) | 0.1203 | 0.643 (0.356–1.161) | 0.1427 |
| 06 | 174 | 85.6% (79.5–90.5%) | 1.010 (0.630–1.618) | 0.9686 | 0.888 (0.477–1.652) | 0.7071 |
| 07 | 160 | 85.0% (78.5–90.2%) | 0.960 (0.593–1.553) | 0.8673 | 0.660 (0.344–1.265) | 0.2105 |
| 08 | 117 | 76.1% (67.3–83.5%) | **0.538 (0.336–0.864)** | **0.0103** | **0.455 (0.225–0.921)** | **0.0287** |
| 09 | 107 | 71.0% (61.5–79.4%) | **0.415 (0.260–0.662)** | **0.0002** | **0.362 (0.188–0.700)** | **0.0025** |
| 10 | 86 | 87.2% (78.3–93.4%) | 1.155 (0.593–2.248) | 0.6718 | 0.911 (0.384–2.159) | 0.8315 |
| 11 | 76 | 69.7% (58.1–79.8%) | **0.390 (0.229–0.664)** | **0.0005** | **0.537 (0.260–1.107)** | **0.0921** |
| 12 | 66 | 40.9% (29.0–53.7%) | **0.117 (0.069–0.200)** | **<0.0001** | **0.193 (0.086–0.432)** | **<0.0001** |
| 13 | 60 | 81.7% (69.6–90.5%) | 0.755 (0.380–1.499) | 0.4211 | 0.434 (0.169–1.116) | 0.0832 |
| 14 | 54 | 81.5% (68.6–90.8%) | 0.745 (0.364–1.527) | 0.4218 | 0.826 (0.305–2.241) | 0.7077 |
| 15 | 52 | 67.3% (52.9–79.7%) | **0.349 (0.188–0.645)** | **0.0008** | 0.642 (0.258–1.596) | 0.3404 |
| 16 | 43 | 90.7% (77.9–97.4%) | 1.651 (0.578–4.718) | 0.3490 | 1.750 (0.456–6.714) | 0.4144 |
| 17 | 18 | 83.3% (58.6–96.4%) | 0.847 (0.241–2.976) | 0.7955 | 2.325 (0.44312.198) | 0.3186 |
| 18 | 13 | 38.5% (13.9–68.4%) | **0.106 (0.034–0.330)** | **0.0001** | **0.108 (0.024–0.493)** | **0.0041** |
| 19 | 7 | 42.9% (9.9–81.6%) | **0.127 (0.028–0.576)** | **0.0075** | **0.077 (0.007–0.845)** | **0.0359** |

**Table S6 Clinicopathological factors associated with %OCP-success (with the institution factor included in multivable analysis)**

| | Univariable (n=2573) | | Multivariable (n=2572) | |
|---|---|---|---|---|
| | Odds (95% CI) | P-value | Odds (95% CI) | P-value |
| DNA integrity (intermediate/high) | **0.210 (0.167–0.265)** | **<0.0001** | **0.195 (0.151–0.251)** | **<0.0001** |
| DNA integrity (low/high) | **0.006 (0.004–0.010)** | **<0.0001** | **0.007 (0.004–0.012)** | **<0.0001** |
| Sample-submitted institution (ID02-19/ID01)† | **(provided in Table S4)** | **<0.0001** | **(provided in Table S4)** | **<0.0001** |
| Sex (female/male) | **1.198 (1.005–1.427)** | **0.0441** | 1.053 (0.827–1.341) | 0.6765 |
| Age (>50 years/≤50 years) | 0.906 (0.703–1.168) | 0.4484 | 1.256 (0.894–1.764) | 0.1892 |
| Specimen type (surgical/biopsy) | **1.542 (1.305–1.821)** | **<0.0001** | **1.796 (1.392–2.319)** | **<0.0001** |
| Site of obtained specimen (metastatic/primary) | 1.054 (0.834–1.334) | 0.6584 | 1.002 (0.713–1.408) | 0.9906 |
| Histology (non-adenocarcinoma/adenocarcinoma) | **0.663 (0.530–0.829)** | **0.0003** | 0.777 (0.555–1.088) | 0.1425 |
| Primary tumor site (non-colorectal/colorectal) | **0.580 (0.486–0.692)** | **<0.0001** | **0.674 (0.517–0.878)** | **0.0035** |
| FFPE-sample storage period (≥4 years/<4 years)‡ | **0.215 (0.158–0.290)** | **<0.0001** | **0.376 (0.249–0.568)** | **<0.0001** |
| Previous chemotherapy (received/not received) | **1.252 (1.017–1.541)** | **0.0338** | 1.061 (0.785–1.434) | 0.6988 |
| Previous radiotherapy (received/not received) | 0.933 (0.562–1.550) | 0.789 | 0.774 (0.385–1.553) | 0.4706 |

†Details are listed in Table S4.

‡One case with no records was excluded from univariable analysis.

**Table S7 Clinicopathological factors associated with %combined-success (with the institution factor included in multivariate analysis)**

| | Univariable (n=2573) | | Multivariable (n=2572) | |
|---|---|---|---|---|
| | Odds (95% CI) | P-value | Odds (95% CI) | P-value |
| DNA integrity (intermediate/high) | **0.206 (0.137–0.309)** | **<0.0001** | **0.214 (0.141–0.326)** | **<0.0001** |
| DNA integrity (low/high) | **0.009 (0.006–0.013)** | **<0.0001** | **0.011 (0.007–0.017)** | **<0.0001** |
| Sample-submitted institution (ID02-19/ID01)† | **(provided in Table S5)** | **<0.0001** | **(provided in Table S5)** | **0.0002** |
| Sex (female/male) | **1.246 (1.000–1.552)** | **0.0495** | 1.144 (0.837–1.565) | 0.3983 |
| Age (>50 years/≤50 years) | 0.961 (0.703–1.313) | 0.8014 | 1.451 (0.938–2.243) | 0.0941 |
| Specimen type (surgical/biopsy) | **1.667 (1.356–2.049)** | **<0.0001** | **1.482 (1.064–2.065)** | **0.0200** |
| Site of obtained specimen (metastatic/primary) | 0.954 (0.717–1.268) | 0.7433 | 0.802 (0.524–1.230) | 0.3120 |
| Histology (non-adenocarcinoma/adenocarcinoma) | **0.709 (0.543–0.925)** | **0.0114** | 1.107 (0.727–1.685) | 0.6365 |
| Primary tumor site (non-colorectal/colorectal) | **0.495 (0.393–0.623)** | **<0.0001** | **0.606 (0.429–0.856)** | **0.0044** |
| FFPE-sample storage period (≥4 years/<4 years)‡ | **0.241 (0.178–0.325)** | **<0.0001** | **0.444 (0.284–0.695)** | **0.0004** |
| Previous chemotherapy (received/not received) | **1.439 (1.099–1.884)** | **0.0082** | 1.264 (0.841–1.898) | 0.2594 |
| Previous radiotherapy (received/not received) | 1.099 (0.572–2.112) | 0.7762 | 0.849 (0.342–2.104) | 0.7232 |

†Details are listed in Table S5.

‡One case with no records was excluded from univariable analysis.

**Figure S1 OCP and CE-IVD success rates in biopsy and surgical samples**

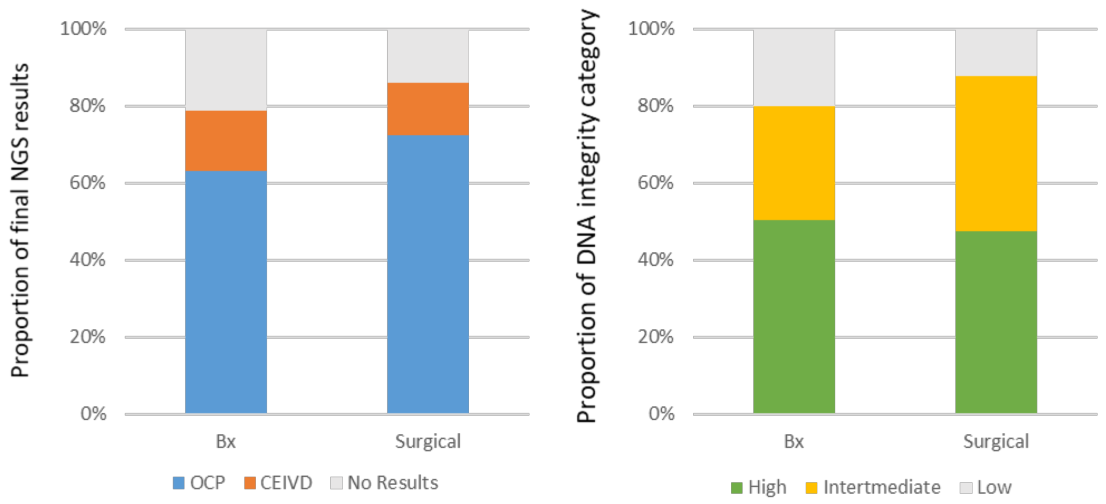

Supplement: Supplementary file 1 — Supporting information. [file PIN-70-932-s001.pdf]
